# Optimized Harvest Management Strategy Based on Latent Loss and Antioxidant Enzyme Activity

**DOI:** 10.3390/foods14071197

**Published:** 2025-03-28

**Authors:** Yujia Wang, Wenfu Wu, Jie Xu, Ming Gao, Zidan Wu, Rui Wang, Houqing Liu

**Affiliations:** 1 School of Biological and Agricultural Engineering, Jilin University, Changchun 130022, China; 2 Wilmar (Shanghai) Biotechnology Research & Development Center Co., Ltd., Shanghai 200137, China

**Keywords:** latent loss, timely harvest, antioxidant enzyme activity, harvest period

## Abstract

Timely harvesting is a crucial aspect of agricultural production, requiring the comprehensive evaluation of multiple factors to optimize yield and quality while minimizing economic losses and resource waste. This study systematically investigates the latent loss and changes in antioxidant enzyme activity in maize and soybean (*Glycine max*) to refine harvest management strategies. The findings reveal that latent loss is a universal phenomenon across different maize and soybean varieties, with maize exhibiting a dry matter loss of up to 12.1036% and soybean reaching 5.5742%. As the harvest progresses, the 1000-grain weight at standard moisture initially increases post-maturity, stabilizes gradually, and may eventually decline, while antioxidant enzyme activity follows a similar trajectory, demonstrating inter-varietal differences. Based on these findings, this study innovatively relates to latent loss and antioxidant enzyme activity. The optimal harvest period for each grain variety is determined step by step to maximize economic benefits, enhance market competitiveness, and ensure the safety and efficiency of grain production.

## 1. Introduction

Postharvest grain loss is a global issue [1,2], with harvest loss being the most prevalent type of food loss worldwide [3]. Numerous studies have investigated the optimal harvest period for grains, providing valuable guidance for improving farmers’ grain yield and quality [4,5,6]. Research on loss reduction and quality assurance in rice production began earlier in foreign countries [7,8]. In China, active efforts have been made to explore the 5T management method [9] and the six-step precision control technology for fresh rice [10], incorporating the concept and technology of timely harvest to establish a new pathway for quality preservation and loss reduction [11]. High-quality, high-yield grain depends not only on prenatal variety optimization, high-quality base selection, and advanced cultivation techniques [12,13], but also on refined harvest management strategies.

Using high-quality rice as a representative species, the research team identified and confirmed the existence and magnitude of “latent loss” while exploring innovations in timely rice harvest management. In 2019, experiments on three rice varieties at the Jilin experimental base revealed latent dry matter loss. Analyzing the relationship between 1000-grain dry matter weight and days after heading showed that delayed harvest resulted in a 3.47% latent dry matter loss [14]. In 2020, the latent loss of dry matter in rice was further validated at the same experimental base, where tests on 18 rice varieties confirmed the presence of latent loss, with delayed harvest leading to a maximum dry matter loss of 3.53% [15]. In the same year, harvest data from six rice varieties, collected using the 5T Smart Farm Management System through the unit area method, were subjected to linear fitting analysis. The results indicated an overall yield decline between 55 and 75 days after heading, with delayed harvest causing a significant latent loss of 7.16% [16]. The findings demonstrated that rice grain dry matter peaks at a specific stage of maturity before declining over time, a phenomenon termed latent loss. Latent loss primarily refers to indirect quantitative losses occurring post-production, which significantly impact grain quality through metabolic consumption, physical grain loss, and other forms of food loss. By leveraging the latent loss phenomenon, the research team determined the optimal harvest period for rice to facilitate timely harvest management. However, since the study focused exclusively on a single grain type, these findings do not necessarily generalize to all other grain varieties.

It is important to note that latent loss should not be the sole criterion for timely harvest management research. It must be applied flexibly, taking into account the climatic conditions of the year and local environmental factors. Studies have shown that when plants face stress, they produce large quantities of reactive oxygen species (ROS), which can cause oxidative damage to cells [17,18]. Antioxidant enzymes such as SOD, POD, and CAT play essential roles in scavenging excess free radicals, thereby enhancing plant resistance to stress [19,20]. Many metabolic processes in organisms are enzymatic reactions, which are closely linked to growth, maturation, and aging [21]. The activity of antioxidant enzymes reflects the growth status of plants and thus is often used for variety identification and breeding. However, its application in assessing or judging the growth status of grain is relatively rare.

This study used major grain species such as maize and soybean as experimental materials to investigate latent losses during the harvest process. By examining the latent losses of grain and the changes in antioxidant enzyme activity throughout the entire harvest process, the optimal harvest time was determined. The research aimed to explore timely harvest management strategies for grains, providing a reference for understanding the principles of timely harvest and offering a scientific basis for future grain harvest management. Ultimately, the goal is to reduce grain loss and contribute to food security.

## 2. Materials and Methods

### 2.1. Plot Location and Test Materials

The study area is located in Jilin Province, China, situated in the central part of Northeast China, bordered by Liaoning Province, the Inner Mongolia Autonomous Region, and Heilongjiang Province. This region has a temperate continental monsoon climate, with the key crop growth period typically extending from spring to autumn. Jilin Province is a major commodity grain production base in China, consistently ranking first in per capita grain possession, commodity rate, grain transfer, and maize exports [22,23]. The primary crops in the region are maize and soybean, which are traditional staple crops [24,25].

In this experiment, four maize varieties—XY335, FM985, JD31, and KX3564—were selected, along with four soybean varieties—JY52, JL16, JY47, and JL8. The characteristics of the selected varieties are shown in the Table 1.

**Figure 1:**
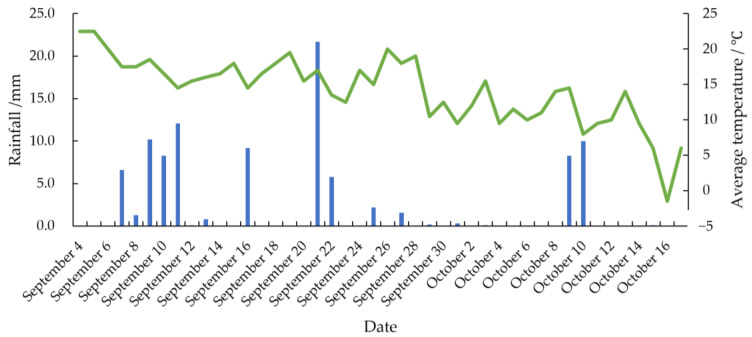
Temperature and rainfall during the experiment.

The experiment was conducted at the maize and soybean experimental base in Gongzhuling City, Changchun City, Jilin Province (longitude: 124.82°, latitude: 43.53°, altitude: 214 m). The experimental sampling period for maize and soybean spanned from 4 September 2021, to 17 October 2021, covering part of the grain filling period through to the full maturity period. A combination of organic and inorganic fertilizers was applied as basal fertilizer. Integrated pest management (IPM) was implemented, combining agricultural and chemical control strategies. The first pesticide application on 1 July 2021 included 40% Syringa tebuconazole (40 mL/666.7 m^2^) and 20% chlorantraniliprole (5 mL/666.7 m^2^). The second application on 29 July 2021 used 30% boscalid and fludioxonil (40 mL/666.7 m^2^) and 40% chlorantraniliprole and thiamethoxam (8 g/666.7 m^2^). The temperature and amount of rainfall during the experiment are shown in the Figure 1.

### 2.2. Collection of Experimental Samples

The following steps were followed during the experiment. First, a flat and fertile field of suitable size was selected as the experimental plot for each maize or soybean variety. Sampling was performed using the 5-point sampling method. The process is as follows: divide the research area into four quadrants, determine the central point, select one sampling point at the central point and four corners (northwest, northeast, southwest, and southeast), totaling five points, and then collect samples at these points according to the standard method to ensure that the sampling covers the whole area and is representative. Every day at noon, samples were collected at fixed points within the designated experimental area, with random sampling conducted (avoiding the field edges). After threshing, the samples from each experimental area were placed in sealed bags and clearly labeled. Subsequently, various indexes, including water content, 1000-grain weight, peroxidase (POD), superoxide dismutase (SOD), and catalase (CAT), were determined.

### 2.3. Data Processing and Statistical Analyses

This research employed major cereal crops, including maize and soybeans, as experimental subjects to explore latent losses during the harvesting process. By analyzing the extent of latent losses and variations in antioxidant enzyme activity throughout the harvesting period, the optimal harvest timing was identified. The various indicators were measured and calculated as follows. Data were analyzed using two-way ANOVA with species, variety, and time as fixed factors, followed by Tukey’s post hoc tests to compare treatment means. Analyses were conducted for both species combined and separately.

#### 2.3.1. Determination of Standard Moisture 1000-Grain Weight

The moisture content (WH_2_O) of grains was determined using the accurate moisture determination method [26,27]. Approximately 500 grains were randomly selected using the quartering method, the complete grains were selected and weighed (*m_t_*), and the number of complete grains was recorded (N). The dry basis 1000-grain weight ($M_{0}$) was then calculated using Formula (1), with the result accurate to 0.0001 g. All test results were repeated three times to obtain the average value. The formula is as follows:

(1) $$M_{0}\left(g\right)=m_{t}\times 10\times \left(100-W_{H_{2}O}\right)÷N$$

Finally, the dry basis 1000-grain weight was converted into the standard moisture 1000-grain weight (M), with the water content set at 15.0%.

(2) $$M\left(g\right)=M_{0}÷\left(1-0.15\right)$$

#### 2.3.2. Latent Dry Matter Loss and Loss Rate

Statistical methods were employed in this experiment. If the difference between the middle value and the data on either side of the three data points exceeded 5%, the “average method” was used to replace the “abnormal data”. After processing, the data were analyzed and operated upon.

To analyze the direct influence of standard moisture 1000-grain weight and post-harvest time, both linear and nonlinear regression analyses were performed using all the obtained data. Based on the optimal harvest date, a linear regression equation was established between the standard moisture 1000-grain weight and the number of days after harvest, and the R^2^ value was calculated. The optimal harvest date is defined as the date when the standard moisture 1000-grain weight of the grain reaches its maximum during production.

In this paper, the latent loss of dry matter (∆m) in grain is defined as the weight change between the standard moisture 1000-grain weight on the optimal harvest day ($m_{1}$) and the standard moisture 1000-grain weight on the average harvest day ($m_{0}$), as shown in Formula (3). The latent loss rate of dry matter (ULR) in grain is defined as the ratio of the latent loss of dry matter to the standard moisture 1000-grain weight on the optimal harvest day, as shown in Formula (4).

(3) $$∆m=m_{1}-m_{0}$$

(4) $$URL=\left(m_{1}-m_{0}\right)÷m_{1}\times 100\%$$

#### 2.3.3. Determination of Antioxidant Enzyme Activity

We chose a representative sample weighing 100 g and eliminated impurities. We ground the samples into powder using a mini plant grinder, sealed all ground samples in self-sealing bags, and stored them at 4 °C. While grinding, we took precautions to prevent overloading or overheating the grinder. Next, we measured the necessary sample proportion in homogenizing tubes and homogenized it using a mechanical homogenizer at a speed of 10,000 to 15,000 rpm. We homogenized the sample under ice-water bath conditions, performing 15 s of homogenization followed by a 30 s pause, repeated 3 to 5 times to achieve the required homogenate. During the experiments, we adhered rigorously to the instructions from the kit manufacturers (from Nanjing Jiancheng Bioengineering Institute, Nanjing, China) to ensure accurate and reliable results.

##### SOD Enzyme Activity

This study employs the Total Superoxide Dismutase (T-SOD) test kit to measure enzyme activity. The kit utilizes the xanthine oxidase method [26,27] (hydroxylamine method) to determine SOD activity, following the specific procedures outlined in the T-SOD test kit instruction manual. In this method, the reaction system involving xanthine and xanthine oxidase generates superoxide anion radicals (O^2−^·). These radicals oxidize hydroxylamine to form nitrite, which, under the action of a color developer, exhibits a purple-red color. The absorbance of this color is measured using a visible light spectrophotometer. Given the parabolic relationship between the percentage inhibition of the enzyme and enzyme activity, the optimal sample concentration should yield a percentage inhibition between 45% and 55%. The inhibition rate of absorbance is calculated using Formula (5), and the SOD activity in the sample is determined using Formula (6) based on the inhibition rate.

(5) $$R=\left(A_{1}-A_{0}\right)÷A_{1}\times 100\%$$

Here, R is the oxidation inhibition rate, %; $A_{1}$ is the control tube absorbance; $A_{0}$ is the assay tube absorbance.

(6) $$U=R÷50\%\times \left(V_{1}÷V_{0}\right)÷n$$

Here, U is the total SOD activity, U/mL; R is the oxidation inhibition rate, %; $V_{1}$ is the total volume of reaction solution, mL; $V_{0}$ is the sampling volume, mL; and n is the homogenate concentration, g/mL. In this reaction system, the enzyme corresponding to the SOD inhibition rate reaching 50% amounts to one SOD activity unit (U).

##### CAT Enzyme Activity

This study utilizes the ammonium molybdate method [28,29] to determine CAT activity, following the specific procedures outlined in the Nanjing CAT assay kit instructions. The decomposition of H_2_O_2_ by catalase is rapidly halted by the addition of ammonium molybdate. The residual H_2_O_2_ reacts with ammonium molybdate to form a yellowish complex, and the CAT activity is quantified by measuring the absorbance change at 405 nm. The CAT activity in the sample is calculated using Formula (7).

(7) $$U=\left(A_{1}-A_{0}\right)\times 271÷V_{s}÷T÷n$$

Here, U is CAT viability, U/mL; $A_{1}$ is the absorbance of control tube; $A_{0}$ is the absorbance of assay tube; $V_{S}$ is the sampling volume, 0.1 mL; T is the reaction time, 60 s; n is the concentration of homogenate, g/mL; the value 271 corresponds to the reciprocal of the slope, which is a constant and is applied directly in the calculations.

##### POD Enzyme Activity

Peroxidase (POD) is a class of oxidative enzymes widely found in various animals, plants, and microorganisms. These enzymes directly oxidize phenolic or amine compounds using H_2_O_2_ as the electron acceptor, thereby eliminating hydrogen peroxide and reducing phenolic amine toxicity. The enzyme activity was determined by measuring the change in absorbance at 420 nm, based on the principle that POD catalyzes the reaction of hydrogen peroxide [30]. The POD activity in the sample was calculated using Formula (8).

(8) $$U=\left(A_{0}-A_{1}\right)÷\left(12\times d\right)\times V_{R}÷V_{S}÷T÷W÷V_{ST}\times 1000$$

Here, U is the POD activity, U/g; $A_{1}$ is the absorbance of the control tube; $A_{0}$ is the absorbance of the assay tube; $V_{R}$ is the total volume of the reaction system, 4 mL; $V_{S}$ is the volume of the sample, 0.1 mL; T is the reaction time, 30 min; W is the fresh weight of the tissue, g; $V_{ST}$ is the total volume of the homogenate, mL.

## 3. Results

### 3.1. Determination Results of the Standard Moisture 1000-Grain Weight and Moisture Content of Grain

**Figure 2:**
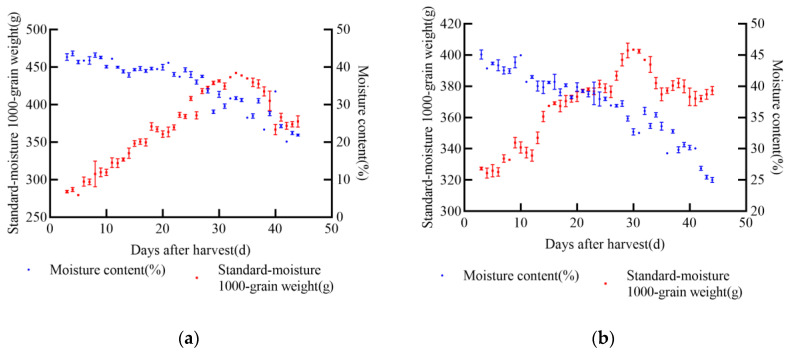

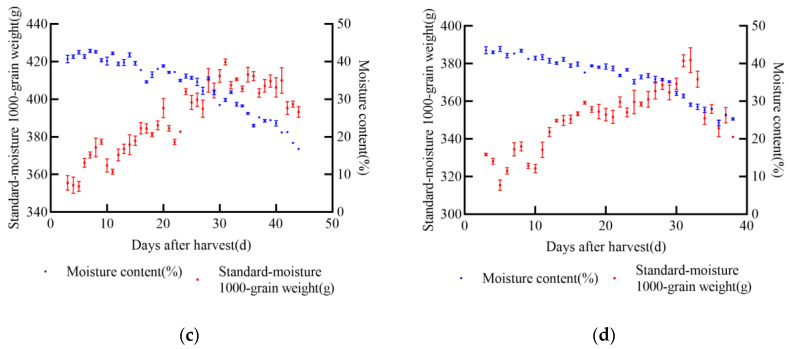
Relationship between standard moisture 1000-grain weight, moisture content, and post-harvest days of maize in 2021, (**a**) JD31, (**b**) FM985, (**c**) XY335, (**d**) KX3564.

In 2021, the experiment was conducted in Gongzhuling City, Changchun City, Jilin Province. The selected maize varieties were XY335, FM985, JD31, and KX3564. Uniform and fully matured grains from the middle of each ear were selected for the experiment. Two indexes, namely 1000-grain weight and moisture content, were determined, and a standard moisture 1000-grain weight model was established. During the wax-ripening stage of maize, dry matter accumulation reaches its peak, after which it either stabilizes or declines, indicating latent loss, as shown in Figure 2. The changing trends of standard moisture 1000-grain weight and moisture content varied across the four varieties.

**Figure 3:**
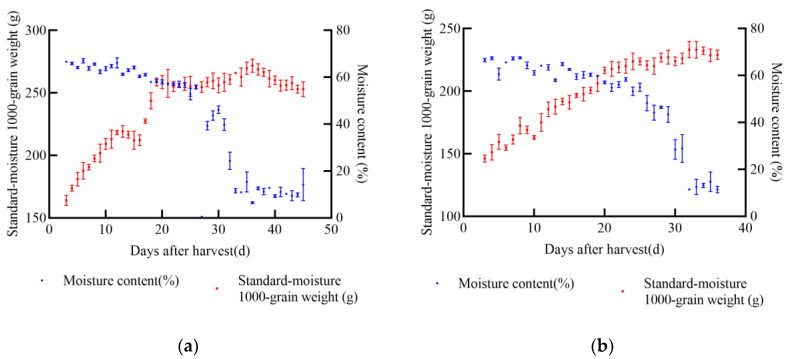

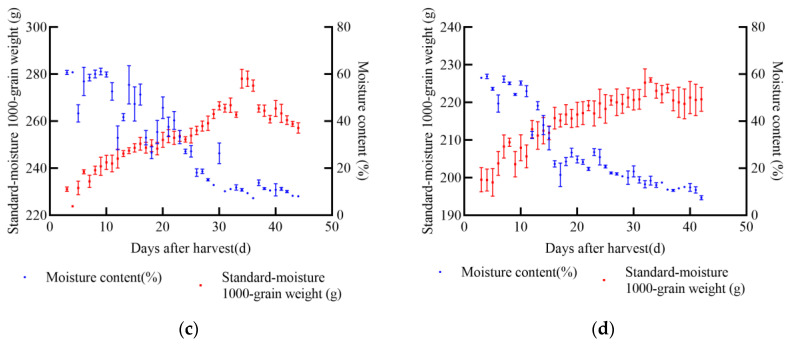
Relationship between standard moisture 1000-grain weight, moisture content, and days after harvest of soybean in 2021, (**a**) JY52, (**b**) JL16, (**c**) JY47, (**d**) JL8.

In 2021, experiments were conducted at the soybean experimental base in Gongzhuling City, Changchun City, Jilin Province. The selected soybean varieties were JY52, JL16, JY47, and JL8. Uniform and fully matured soybean seeds were chosen for the experiments. Two indexes, namely 1000-grain weight and moisture content, were determined, and a standard moisture 1000-grain weight model was established. From the onset of sampling, the standard moisture 1000-grain weight of soybean increased with the passage of time. According to the data from the four soybean varieties, the maximum increase in 1000-grain weight was approximately 104.74 g for JY52, while the minimum increase was about 27.24 g for JL8 during the entire harvest process, as shown in Figure 3. Once the soybean seeds reached maturity, the standard moisture 1000-grain weight stabilized at its peak and did not increase further. After this point, some varieties exhibited notable latent loss.

As shown in the Table 2, the overall model is statistically significant (F = 726.554, *p* < 0.001), demonstrating an excellent fit and explaining 94.9% of the variance in the dependent variable Standard Moisture 1000-Grain Weight (R^2^ = 0.949). Both Variety (F = 760.989, *p* < 0.001; partial η^2^ = 0.945) and Days After Harvest (F = 379.131, *p* < 0.001; partial η^2^ = 0.549) exerted significant effects on Standard Moisture 1000-Grain Weight, highlighting substantial differences in weight across varieties and over time. Notably, Variety exhibited an exceptionally high effect size, underscoring its role as the primary determinant of weight variation. Additionally, Days After Harvest demonstrated a robust temporal influence. The small error term further confirms the model’s strong explanatory power and reliability.

### 3.2. Determination Results of Grain Antioxidant Enzyme Activity

#### 3.2.1. Determination Results of POD

**Figure 4:**
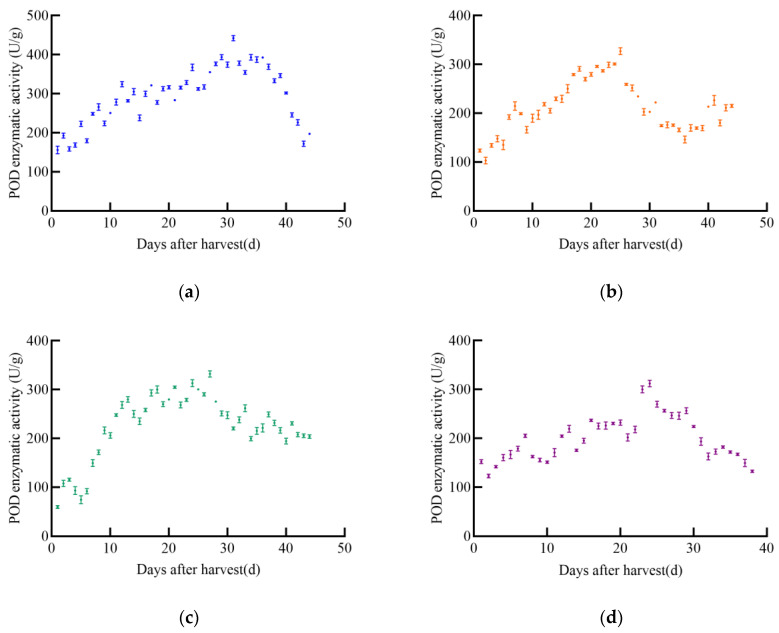
POD enzyme activity curve of maize, (**a**) JD31, (**b**) FM985, (**c**) XY335, (**d**) KX3564.

With the increase in post-harvest time, the overall trend of POD enzyme activity in different maize varieties initially increased and then decreased, as shown in Figure 4. The POD enzyme activity of JD31 increased slowly, reaching a maximum value of 442.2 U/g on day 31, after which it declined rapidly. The POD enzyme activity of FM985 increased almost linearly, reaching a maximum value of 327.3 U/g on day 25, then gradually decreased but increased again on day 40. The POD enzyme activity of XY335 showed a steady increase, reaching a maximum value of 332.2 U/g on day 27, followed by a slow decrease. The POD enzyme activity of KX3564 increased with post-harvest time but fluctuated significantly, reaching a maximum value of 312.2 U/g on day 24, and then decreased slowly.

**Figure 5:**
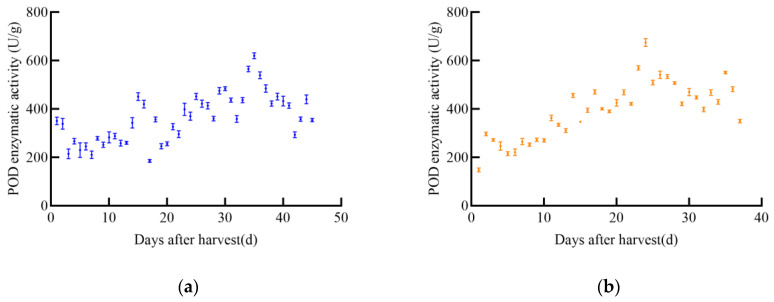

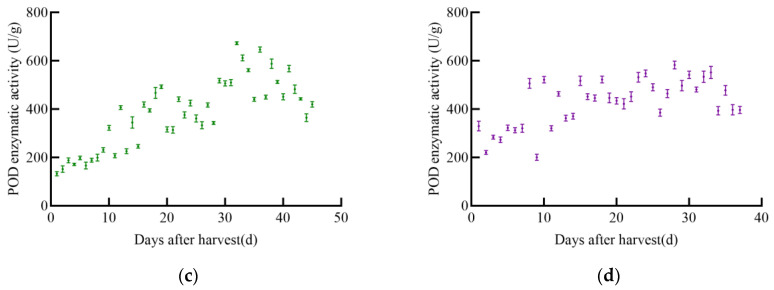
POD enzyme activity profile of soybean, (**a**) JY52, (**b**) JL16, (**c**) JY47, (**d**) JL8.

As shown in Figure 5, POD enzyme activity in soybeans changed with increasing harvest time, generally showing an initial increase followed by a slow decrease. The POD enzyme activity of the four soybean varieties varied significantly. The POD activity of JY52 increased slowly and fluctuated greatly, reaching a peak of 620.2 U/g on day 35 post-harvest, after which it declined. The POD enzyme activity of JL16 increased slowly with fluctuations, peaking at 675.2 U/g on day 34, then decreased immediately. The POD enzyme activity of JY47 increased with post-harvest time, fluctuating to 672.8 U/g on day 32, then began to decrease slowly. The POD enzyme activity of JL8 showed insignificant fluctuations over time, with a peak enzyme activity of 583.3 U/g on day 28.

#### 3.2.2. Determination Results of SOD

**Figure 6:**
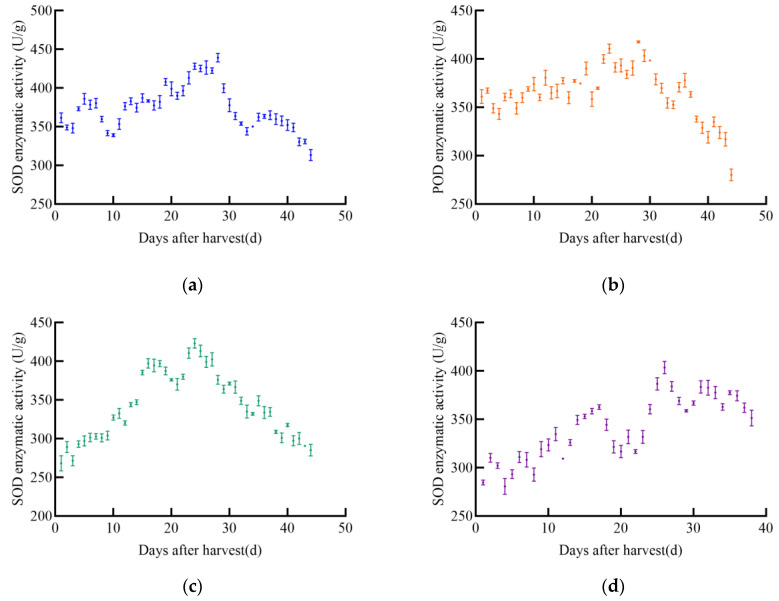
SOD enzyme activity profile of maize, (**a**) JD31, (**b**) FM985, (**c**) XY335, (**d**) KX3564.

As the post-harvest time increases, the overall trend of SOD enzyme activity first increases and then decreases in different maize varieties, as shown in Figure 6. The SOD enzyme activity of JD31 shows an upward trend, reaching a peak of 439.2 U/g on the 28th day after harvest, then rapidly declining. Although there are slight increases during the decline, the overall trend is still downward. The SOD enzyme activity of FM985 shows a slow upward trend with little fluctuation, reaching a maximum value of 417.7 U/g on the 28th day and then gradually decreasing. The SOD enzyme activity of XY335 shows an upward–downward–upward trend, reaching a maximum value of 423.2 U/g on the 24th day after harvest and then gradually decreasing. The early trend of SOD enzyme activity in KX3564 is similar to that of XY335, showing an upward–downward–upward trend, reaching a maximum value of 403.6 U/g on the 26th day after harvest and then declining.

**Figure 7:**
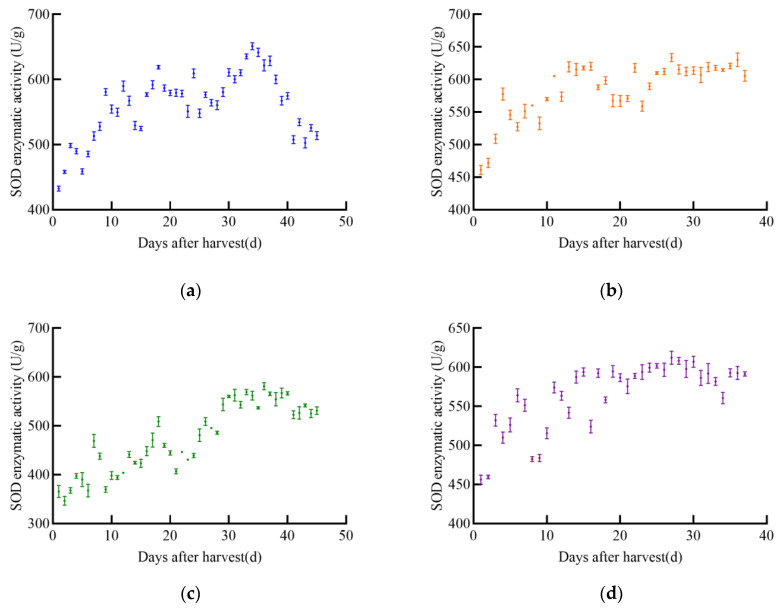
SOD enzyme activity profile of soybean, (**a**) JY52, (**b**) JL16, (**c**) JY47, (**d**) JL8.

As shown in Figure 7, the SOD enzyme activity of soybeans changes with the increase in harvest time, and the trends vary significantly among the four varieties. The SOD enzyme activity of JY52 shows an upward–downward–upward trend, reaching a maximum value of 651.2 U/g on the 34th day and then gradually decreasing. The overall trend of SOD enzyme activity in JL16 is a rapid increase followed by a slow increase, reaching a maximum value of 633.7 U/g on the 27th day. The trend of SOD enzyme activity in JY47 is similar to that of JL16, showing a gradual increase with significant fluctuations, reaching a stable upward phase around the 30th day, with a peak value of 581.1 U/g on the 36th day. The SOD enzyme activity of JL8 first increases with little fluctuation, maintaining a stable range around 20 days after harvest, with a maximum value of 612.3 U/g.

#### 3.2.3. Determination Results of CAT

**Figure 8:**
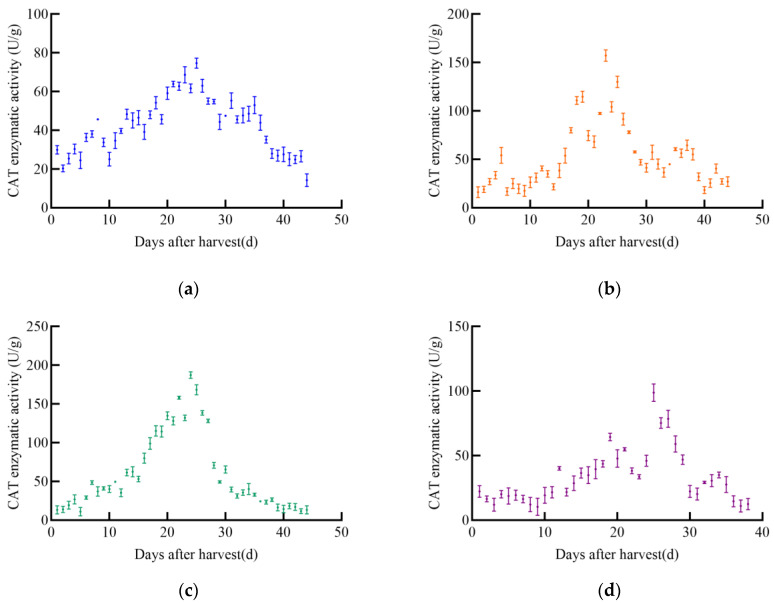
CAT enzyme activity curve of maize, (**a**) JD31, (**b**) FM985, (**c**) XY335, (**d**) KX3564.

With the increase in post-harvest time, the catalase (CAT) enzyme activity of different maize varieties exhibited significant changes, generally following an initial increase followed by a decrease, as illustrated in Figure 8. The CAT enzyme activity of JD31 peaked at 74.7 U/g on the 25th day and then gradually declined. Similarly, FM985 reached its maximum CAT activity of 157.2 U/g on the 23rd day before gradually decreasing. The CAT enzyme activity of XY335 initially increased linearly, peaking at 187.4 U/g on the 24th day, after which it gradually declined. In contrast, KX3564 showed an insignificant increase in CAT activity, reaching a maximum of 98.9 U/g on the 25th day before decreasing.

**Figure 9:**
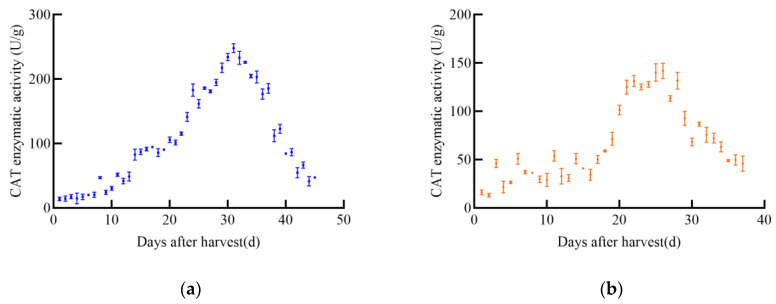

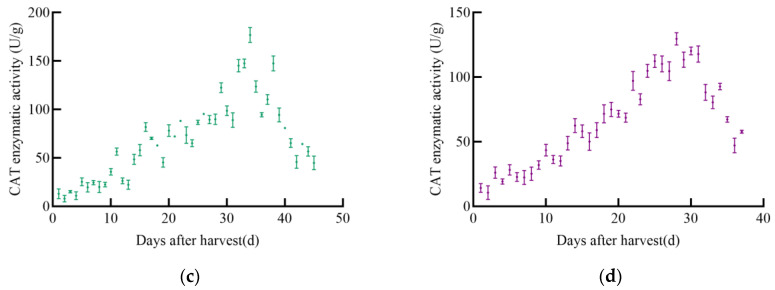
CAT enzyme activity curve of soybeans, (**a**) JY52, (**b**) JL16, (**c**) JY47, (**d**) JL8.

As shown in Figure 9, CAT enzyme activity in soybeans also changed significantly with increasing post-harvest time, following a general trend of increase followed by decrease. The CAT enzyme activity of JY52 increased to a maximum of 248.1 U/g on the 31st before declining. JL16’s CAT activity increased and then stabilized for a period before rapidly increasing to a peak of 141.9 U/g on the 26th, followed by a decline. Both JY47 and JL8 exhibited similar trends, with a slow increase in CAT activity followed by a rapid decrease. JY47 reached a maximum CAT activity of 177.0 U/g on the 34th day, while JL8 peaked at 129.7 U/g on the 28th.

## 4. Discussion

### 4.1. The Existence of Latent Loss and the Calculation of Latent Loss Rate

**Figure 10:**
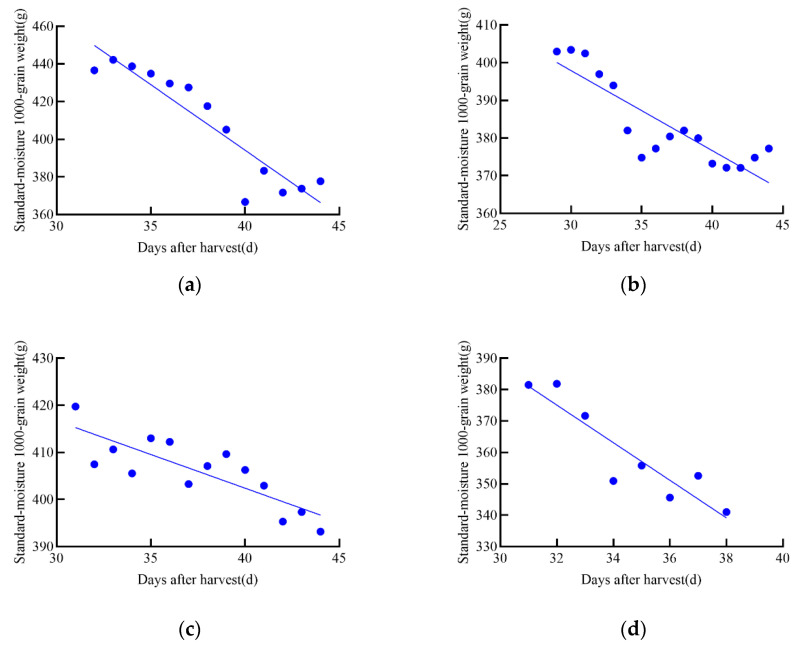

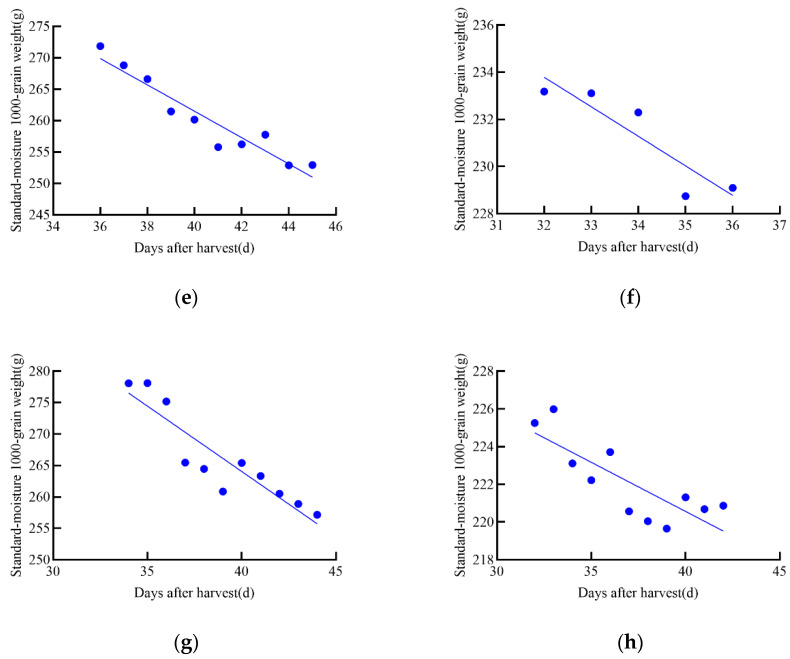
Linear regression equation of standard moisture 1000-grain weight for maize and soybean in 2021. (**a**–**d**) represent the four maize varieties in the 2020 experiment: (**a**) JD31, (**b**) FM985, (**c**) XY335, (**d**) KX3564. (**e**–**h**) represent the four soybean varieties in the 2020 experiment: (**e**) JY52, (**f**) JL16, (**g**) JY47, (**h**) JL8.

During the tracking and measurement of growth data for maize and soybean seeds, it was observed that dry matter decreased in the later stages of growth, further confirming the latent loss of dry matter during the growth process of maize and soybean, as shown in Figure 10. This finding is consistent with previous studies [15], indicating that crops may undergo natural depletion or loss of dry matter during the later stages of maturation.

To analyze the relationship between standard moisture 1000-grain weight and post-harvest time, both linear and nonlinear regressions were performed using the obtained data. To identify the best model for each relationship, first-order and second-order models were constructed, and the model with the highest R^2^ value was selected. The best harvest date was then chosen as the reference point, and a linear regression equation was established between the standard moisture content of 1000 grains weight (g) and the days after harvest (days), as shown in Table 3. The best harvest date corresponds to the day when the dry matter weight of the grain reaches its maximum during growth.

Assuming that the loss of maize and soybeans on the optimal harvest day is 0, latent loss weight and latent loss rate were calculated based on the actual harvest day records. The data indicate that the later the harvest, the greater the dry matter loss. In other words, if the grain is not harvested at the optimal time, the grain yield will decrease rather than increase as the harvest time is delayed, resulting in latent losses (Table 4). Specifically, the dry matter loss in maize can reach up to 12.1036%, while in soybean, it can reach 5.5742%. In 2024, the total output of maize and soybean in China reached 294.917 million tons and 23.628 million tons [31], respectively. By applying the average latent loss rate of dry matter, the latent dry matter loss for maize and soybeans was calculated as follows: for maize, 294.917 million tons × 12.1036% = 35.696 million tons; for soybeans, 23.628 million tons × 5.5742% = 1.317 million tons. Furthermore, assuming the average purchase prices of maize and soybeans are 2063 yuan/ton and 3804 yuan/ton, respectively, the economic losses due to latent dry matter losses are approximately 2063 yuan/ton × 35.696 million tons = 73.64 billion yuan for maize and 3804 yuan/ton × 1.317 million tons = 5.01 billion yuan for soybeans.

Furthermore, the results indicate significant varietal differences in dry matter loss rates among different grain varieties. For instance, the dry matter loss rate for the maize variety JD31 is as high as 17.29%, whereas the loss rate for the soybean variety JL8 is only 2.56%. These variations may be attributed to differences in genetic traits, growth environments, and stress resistance capacities among the varieties. Future studies should focus on elucidating the mechanisms underlying dry matter loss in different varieties, particularly under varying climatic conditions, to provide deeper insights into these observed discrepancies.

### 4.2. Determination of the Timely Harvest Period

Numerous studies have investigated the optimal harvest period for grain, which significantly contributes to improving both the yield and quality of farmers’ crops. For instance, the research collaboration group on the production technology system and application theory of high-quality rice in Hunan Province has identified ear moisture content of approximately 21.0% as one of the key diagnostic indicators for determining the appropriate harvest time, based on rice yield and milling quality. Additionally, for practical application and ease of adoption by farmers, the number of days after full heading of rice is used as a criterion for timely harvesting [32]. The assessment of rice moisture content offers a relatively convenient method for determining harvest timing, even when the number of days after heading or accumulated temperature data are unavailable [33]. Furthermore, Zhang Na et al. [34] analyzed the losses associated with traditional post-harvest rice treatment methods and demonstrated that implementing the 5T management method can significantly reduce losses and increase yield. Their study revealed that strict control of factors during each production phase, as part of the 5T management process, can enhance both yield and economic benefits by mitigating hidden losses caused by improper management during harvesting and subsequent operations. At the time of harvest, the number of grains per unit area has been determined. Therefore, selecting the optimal harvest period is crucial for maximizing economic value under the same grain quantity. The study by Dong et al. [35] demonstrated that increased maize yield is associated with enhanced dry matter accumulation and greater carbohydrate storage. Additionally, the improved scavenging capacity of SOD and POD enzymes positively influences grain quality. Similarly, Wang et al. [36] found that elevated activities of SOD, POD, and CAT enzymes significantly reduce the degree of kernel senescence in maize. Although numerous studies have investigated maize and soybean growth curves and antioxidant enzyme activities, limited research has utilized these parameters to determine the optimal harvest time for maize and soybean.

By analyzing the activity changes of antioxidant enzymes (POD, SOD, and CAT), we observed that the activities of these enzymes increased during the grain-filling stage, peaked at maturity, and subsequently declined (Section 3.2). These results indicate that changes in antioxidant enzyme activity can serve as an important indicator for determining whether crops are in a suitable harvest period. During the actual grain harvesting process, the levels of antioxidant enzymes increase over time, while the content of H_2_O_2_ gradually decreases, thereby reducing oxidative damage and preserving grain quality [37,38]. Typically, higher antioxidant enzyme activity suggests that the crops have not yet reached the optimal harvest state. Based on these findings, we determined the best harvest periods for different maize and soybean varieties (Table 5). However, it should be noted that the optimal harvest period may vary due to annual climatic conditions and local environmental factors, necessitating flexible adjustments in practical applications.

## 5. Conclusions

1. Latent Loss of Dry Matter and Its Implications

Latent loss of dry matter is a phenomenon observed across various grain varieties. Throughout the harvesting process, the dry matter accumulation in grains follows a pattern of cumulative growth and deceleration in the early stages, reaching its peak before entering a phase of gradual reduction, where dry matter begins to decrease—a phenomenon referred to as latent loss. Latent losses primarily encompass indirect quantitative losses occurring during the post-production phase, which negatively impact grain quality, including metabolic consumption, grain loss, and other forms of food losses. Significant variations in grain moisture content and standard moisture 1000-grain weight were observed at different harvest times, with notable differences among grain varieties. As the harvest period extended, the moisture content of grains decreased, while the standard moisture 1000-grain weight initially increased and then decreased. Among maize varieties, XY335 exhibited the highest increase in weight, whereas KX3564 exhibited the lowest. Similarly, among soybean varieties, JY52 demonstrated the highest weight increase, whereas JL8 had the lowest.

2. Antioxidant Enzyme Activities and Its Implications

In this study, the activities of POD, CAT, and SOD enzymes were measured throughout the entire harvest period, and a nonlinear regression model was established to describe the changes in enzyme activities. The results revealed that the activities of SOD, POD, and CAT enzymes underwent significant changes as the harvest period progressed Overall, enzyme activity initially increased, peaked, and then decreased, with substantial variations observed among different grain varieties. Based on the changes in dry matter accumulation and enzyme activities during grain growth, the optimal harvest time for different grain varieties was determined. This approach provides a scientific basis for optimizing harvest timing to maximize grain quality and yield.

3. Research significance and future prospect

Agriculture serves as a key driver of economic growth in the region [39], and reducing food loss and waste remains a long-term and complex challenge. This study introduces a novel perspective aimed at establishing guiding principles for timely grain harvest. By integrating the analysis of dry matter loss and antioxidant enzyme activities, this research offers a scientific foundation for improving harvest management practices. Furthermore, it promotes a shift in agricultural production concepts and practices, ultimately contributing to the reduction of grain loss and the enhancement of food security.

This study offers valuable insights into dry matter loss and optimal harvest timing for corn and soybean, yet some limitations exist. The findings rely solely on 2021 data, necessitating future research to validate their stability and generalizability. Additionally, the impacts of climatic factors—such as rainfall, drought, or extreme temperatures—on dry matter loss and antioxidant enzyme activity remain unexplored. Further investigation into these environmental influences, along with the integration of physiological and molecular indicators, could enhance understanding of crop maturation and harvest mechanisms. In addition, this study has limitations in terms of variety selection. While several representative maize and soybean varieties were selected for investigation, these may not fully capture the genetic and phenotypic diversity present across all maize and soybean varieties. Significant variations in dry matter loss, antioxidant enzyme activity, and responses to environmental factors are likely to exist among different varieties. Therefore, future studies should incorporate a broader range of varieties to provide a more comprehensive evaluation of the universality of dry matter loss and optimal harvest timing.

## Figures and Tables

**Table 1:** The characteristics of the selected varieties.

Variety		Mature Period	Spike Type	Grain Color
Maize	JD31	Medium–early maturing	Long tube type	Yellow
	FM985	Medium maturing	Long tube type	Yellow
	XY335	Medium maturing	Long tube type	Yellow
	KX3564	Medium maturing	Long tube type	Yellow
Soybean	JY52	Medium maturing	Round particle type	Yellow
	JL16	Medium maturing	Elliptic granular type	Green
	JY47	Medium maturing	Elliptic granular type	Yellow
	JL8	Early maturing	Elliptic granular type	Green

**Table 2:** Between-subjects effects test. Dependent variable: Standard Moisture 1000-Grain Weight.

Source	Type III Sum of Squares	df	Mean Square	F	*p*	Partial Eta Squared
Corrected Model	1,819,043.574 ^a^	8	227,380.447	726.554	<0.0001	0.949
Intercept	4,626,006.499	1	4,626,006.499	14,781.575	<0.0001	0.979
Variety	1,667,100.477	7	238,157.211	760.989	<0.0001	0.945
Days After Harvest	118,651.998	1	118,651.998	379.131	<0.0001	0.549
Error	97,642.774	312	312.958			
Total	30,851,907.731	321				
Corrected Total	1,916,686.348	320				

a. R^2^ =0.949 (Adjusted R^2^ = 0.948).

**Table 3:** Standard moisture 1000-grain weight models based on a 15% standard moisture content.

Variety		Regression Equation	R^2^	*p*
Maize	JD31	Y = −6.961×X + 672.6	0.8536	<0.0001
	FM985	Y = −2.127×X + 461.8	0.7534	<0.0001
	XY335	Y = −1.430×X + 459.6	0.6784	0.0003
	KX3564	Y = −5.992×X + 566.9	0.8373	0.0014
Soybean	JY52	Y = −2.098×X + 345.4	0.9086	<0.0001
	JL16	Y = −1.306×X + 275.6	0.8780	0.0006
	JY47	Y = −2.080×X + 347.3	0.8317	<0.0001
	JL8	Y = −0.521×X + 241.4	0.6581	0.0024

Y represents the standard moisture content of 1000 grains (g); X represents the number of days after harvest (days).

**Table 4:** Latent loss weight and dry matter loss rate based on a 15% standard moisture content.

Variety		Loss Weight/g	Average	Loss Rate/%	Average
Maize	JD31	76.5710	49.2108	17.2891	12.1036
	FM985	29.7780		7.4821	
	XY335	18.5900		4.4766	
	Kx3564	71.9040		19.1664	
Soybean	JY52	16.7840	14.2262	6.2192	5.5742
	JL16	15.6720		6.7029	
	JY47	18.7200		6.8197	
	JL8	5.7288		2.5551	

**Table 5:** The optimal harvesting period for different varieties of maize and soybean.

Days After Harvest	Maize				Soybean			
	XY335	FM985	JD31	KX3564	JY52	JL16	JY47	JL8
Weight	27–34	27–34	29–36	26–33	33–40	30–35	32–39	29–36
POD	27–34	25–32	31–38	24–31	35–42	24–31	32–39	28–35
CAT	24–31	23–30	25–32	25–32	31–38	26–33	34–41	28–35
SOD	24–31	28–35	28–35	26–33	34–41	27–34	36–43	27–34
Optimal Date	27 September–7 October	28 September–8 October	1 October–11 October	26 September–6 October	5 October–15 October	28 September–8 October	6 October–16 October	28 September–8 October

## Data Availability

The original contributions presented in the study are included in the article, further inquiries can be directed to the corresponding author.
